# Application of Temperature Programmed Oxidation-Infrared Technique in the Analysis of Sulfur Occurrence and Genesis in Phosphate Rock

**DOI:** 10.1155/2022/3255760

**Published:** 2022-07-08

**Authors:** Qiuyuan Xu, Shiyun Tang, Anjiang Tang, Yazhou Tian

**Affiliations:** ^1^College of Resources and Environmental Engineering, Key Laboratory of Karst Georesources and Environment, Ministry of Education, Guizhou University, Guiyang, Guizhou 550025, China; ^2^College of Chemical Engineering, Guizhou Institute of Technology, Guiyang 550003, China

## Abstract

In this study, a temperature programmed oxidation-infrared (TPO-IR) technique was improved and applied in the analysis of sulfur occurrence and genesis in phosphate rock. Phosphate rocks from three regions (KYP, ZJP, and WAP) were selected for the detection of sulfur species by TPO-IR combined with XRD, SEM, EDS, and XPS characterization. TPO-IR results show that the total sulfur contents of the three phosphate rocks were 2.14% for KYP, 1.18% for ZJP, and 1.06% for WAP. In the low-temperature area (<1000°C), TPO-IR detected that both KYP and WAP contain FeS with a characteristic temperature of about 513°C and their contents were 9.22‰ and 0.64‰, respectively. In high-temperature areas (>1000°C), the TPO-IR curves suggest that sulfate is the main sulfur species in the three phosphate rocks. Typically, the characteristic temperature near 1070^o^C belongs to MgSO_4_, and the characteristic temperature near 1290°C belongs to CaSO_4_. Due to the incomplete TPO-IR database of sulfur reference materials at present, it is not possible to assign all sulfur species in high-temperature areas. However, in a sense, this research provides theoretical basis and experimental support for the application of the TPO-IR technique for the detection of sulfur species in other solid minerals.

## 1. Introduction

Due to the increasingly prominent problems of resources and the environment, sulfur (S) has attracted more and more attention. It is commonly found in sulfur-containing minerals, animal remains, and plants. Sulfur is considered to be the fourth largest plant nutrient in the world after N, P, and K, and the basic element for maintaining normal life activities [1]. It has been confirmed that sulfur plays a very important role in the development and evolution of life and the atmosphere, as well as in the differentiation of Earth's core and mantle [2, 3]. In industrial production, natural minerals such as sulfide ore, pyrite [4, 5], arsenopyrite, sulfur-gold ore [6], and coal mine [7–9] contain a lot of sulfur, which will seriously affect the production efficiency. At present, more attention has been paid to the qualitative and quantitative analysis of sulfur in natural minerals [10, 11]. Phosphate rock, for example, is a nonmetallic natural mineral and is of great strategic significance to national industrial development and food security. Generally, the main chemical components of phosphate rock are Ca_5_(PO_4_)_3_F, Ca_5_(PO_4_)_3_Cl, and Ca_5_(PO_4_)_3_OH, and a small amount of sulfur is associated with minerals [12–14]. However, previous studies on sulfur in phosphate rock are less, and the occurrence state and genesis of sulfur in phosphate rock lack understanding due to the low content of sulfur in phosphate rock and the lack of utilization value. Therefore, we developed a temperature programmed oxidation-infrared (TPO-IR) technique to detect the small amounts of sulfur species in phosphate rock, and it is believed that it can provide a theoretical basis and experimental support for the application of the TPO-IR technique in the detection of sulfur species in other solid minerals.

The detection of carbon species by the TPO-IR technique has been relatively mature, including carbon nanomaterials [15], anthracite [16, 17], and other carbon materials [18–20]. The principle of sulfur detection by the TPO-IR technique is similar to that of carbon species detection. It is based on the reaction between sulfur atoms and molecular oxygen occurring in special active sites (such as structural defects and edge S atoms), and thus, according to different types of oxygen reactivity of sulfur compounds, sulfur species are identified.

Some achievements have been made in the analysis and determination of sulfur species in solid minerals, including ultraviolet spectrophotometry (UV) [21], traditional chemical methods (such as coulometric titration [22] and gravimetric method [23]), X-ray photoelectron spectroscopy (XPS) [24, 25], and X-ray absorption near-edge structure (XANES) [26]. However, the disadvantage of UV is that there are many interference factors, especially the accuracy will be significantly reduced when the sample composition is complex. The traditional chemical method is cumbersome, for example, the gravimetric method has to go through many operations including dissolution, titration, filtration, and burning. Furthermore, the detection process is affected by many factors, resulting in error accumulation and time consumption. The coulomb method has the disadvantages of low analysis efficiency, poor flexibility of the experimental process, cumbersome instrument maintenance, and a high failure rate. The XANES method is not only economically cost expensive but also has poor accuracy. Because the content of sulfur in phosphate rock is very low and the composition is very complex, it is difficult to obtain accurate qualitative and quantitative results using these methods.

By comparison, the TPO-IR technique has more advantages in the detection of sulfur species in solid minerals, such as fast analysis speed, low sample consumption, high accuracy, low interference, and high detection limit. In previous works, the TPO-IR technique has been used to analyze sulfur in coal and phosphate rock at low-temperature areas (<1000°C) [27]. In this work, we improved the upper limit of the detection temperature range and increased the exploration of sulfur species in the high-temperature areas (>1000°C) of phosphate rock. Focusing on the application characteristics of the TPO-IR technique, combined with other analysis methods, the sulfur species in minerals were qualitatively and quantitatively detected, and the occurrence and genesis of sulfur in phosphate rock were briefly discussed.

## 2. Experimental

### 2.1. Materials

In this work, 18 sulfur species with known structures were selected to establish a qualitative analysis database through TPO-IR detection, including metal sulfides (e.g., Cu_2_S, MnS, FeS, WS_2_, CoS, ZnS, and Ag_2_S), sulfates (e.g., CrK(SO_4_)·12H_2_O, NiSO_4_·6H_2_O, (NH_4_)2S_2_O_8_, Na_2_SO_3_, H_2_N·NH_2_·H_2_SO_4_, (NH_4_)_2_SO_4_, MnSO_4_, MgSO_4_·7H_2_O, FeSO_4_·7H_2_O, and CaSO_4_·2H_2_O), and organic sulfurs (e.g., C_18_H_29_NaO_3_S). All the materials were used without further purification. Their physical properties are summarized in Table 1.

**Figure 1:**
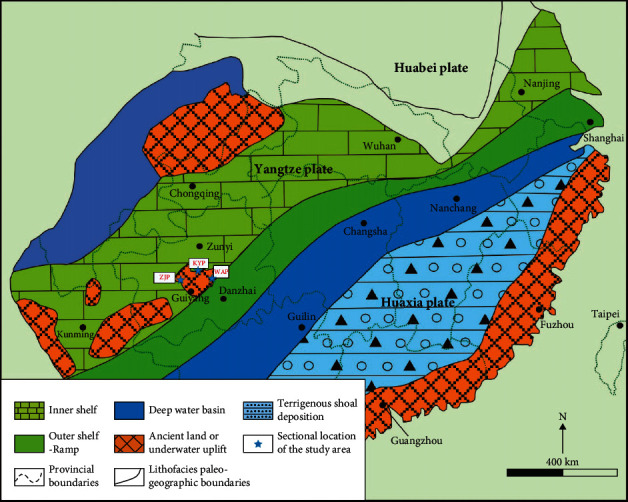
Palaeogeographic map of the Yangtze platform during the depositional period of the Sinian Doushantuo formation (Ref. [28], Fig. 1).

The selected phosphate rock samples were located in Zhijin phosphate (labeled ZJP), Weng'an phosphate (labeled WAP), and Kaiyang phosphate (labeled KYP) in the central Guizhou Province, China.  Figure 1 shows the paleogeographic map of the Yangtze platform during the depositional period of the sampling area [28]. KYP and WAP were formed in the Sinian Doushantuo Dengying formation, which is a well-known super-large phosphate-rich deposit at home and abroad. Phosphate deposits in the Zhijin area are found in the Cambrian Meishucun Niutitang formation. Due to the close interval between Sinian and Cambrian phosphate deposits, the spatial distribution of the phosphate deposits is related to each other, and the inductively coupled plasma mass spectrometry (ICP-MS) detection data show that their chemical components are similar. Taking ZJP as an example, the phosphate rock composition measured by oxide is P_2_O_5_ 28.9%, SiO_2_ 13.13%, MgO 1.89%, CaO 41.2%, and F 2.6%.

### 2.2. Principle of TPO-IR Detection of Sulfur Species

The determination of sulfur species in phosphate rock by TPO-IR is based on the infrared absorption principle of the Lambert–Beer law. Under the pure oxygen atmosphere, the sulfur compounds in the sample are gradually oxidized or decomposed to form SO_2_ by heating the sample in a resistance furnace according to the set heating procedure. Then, the accompanying H_2_O and other gas impurities are filtered and absorbed by the purification system, leaving SO_2_ in the gas system with the carrier gas, and then into the SO_2_ infrared detection cell for detection. Since the characteristic temperature (at this temperature, the conversion rate of the sulfur species to SO_2_ reaches the maximum) of each sulfur species is different, the sulfur species can be judged according to their peak position and the content of sulfur species can be calculated according to the peak intensity.

**Figure 2:**
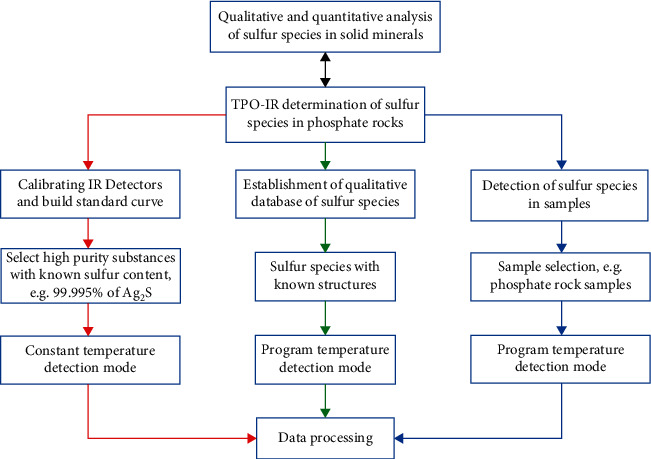
Flow diagram of sulfur species measurement using the TPO-IR method.

The whole TPO-IR detection process is divided into three paths (represented by three different colors, as shown in Figure 2). First, the infrared detector is calibrated, and the standard material used is Ag_2_S with a purity of 99.995%, which is detected at a fixed high temperature of 950°C (as the red path). The corresponding calibration curve is shown in previous works [27], and the standard deviation is about 1.94%. Then, the characteristic temperature was collected in the temperature-programmed detection mode, and the TPO-IR database of sulfur species with known structures was established (as the green path). It lays a theoretical foundation for the identification of unknown sulfur species in phosphate rock. Finally, the TPO-IR technique was used to detect sulfur species in phosphate rock (as the blue path). In this process, the sulfur species in the phosphate rock can be quantitatively calculated by comparing with the standard curve established in the first step, and qualitatively analyzed by comparing the characteristic temperature of the sulfur species with known structures established in the second step. The detailed process will be described in the next section. Furthermore, the first two steps only need to be completed for the first time, and then, the samples can be directly tested. The technique has high sensitivity, simple operation, short time, and accurate detection results.

### 2.3. Experiment of TPO-IR Detection of Sulfur Species

In the TPO-IR process, 99.5% pure O_2_ with a flow rate of 1.80 L/min was used as the carrier gas and oxidizing agent. The TPO device was designed by our group, and the IR detector was the GCS-80 SO_2_ tubular infrared carbon and sulfur analyzer developed by Sichuan Science Instruments Co., Ltd. A detailed description of the device is given in reference [27]. The detection limit of SO_2_ concentration is 1 ppm.

The experimental steps are briefly described as follows: First, powdered samples were weighed about 0.1 g to 0.5 g and put into a ceramic boat which had been calcined at 1500°C for 14400 s. To ensure the complete release of sulfur in the test, the phosphate rock powder was prepared by ball milling the raw ore for 600 s, and drying in an oven at 80°C for 3600 s. Then, it was placed in the temperature zone center of the programmable heating apparatus with a heating rate of 7°C/min. The data from 50°C to 1500°C was collected while the program was running. Finally, the process was manually ended and the heating device was cooled according to the preset program when the SO_2_ was released completely. The formula for calculating the sulfur content in the phosphate rock is as follows:

(1) $$\begin{array}{c} S\%=\frac{M_{t}}{M_{s}}\times 100\%, \end{array}$$

(2) $$\begin{array}{c} S\%=\frac{M_{i}}{M_{s}}\times 1000\%, \end{array}$$

where the S%/S‰ represents the percentage/thousandths of sulfur species in the phosphate rock; “*M*_s_” represents the quality of the phosphate rock powder samples used for TPO-IR detection; “*M*_t_” is the total sulfur mass in the samples; and “*M*_i_” is the mass of the single sulfur species in the samples.

To further analyze and understand the test results of TPO-IR, the samples were characterized by using a Nova Nano SEM 450 thermal field emission scanning electron microscope (SEM) from FEI Company attached to an energy dispersive spectrometer (EDS) from EDAX. An Ultima IV rotating anode X-ray diffractometer (XRD, Rigaku Electric Co., Ltd.) was used to detect and analyze samples before and after the TPO-IR test. Cu Kα (λ= 0.15406 nm) was the radiation source, the goniometer was equipped with a graphite monochromator in the diffraction beam, The X-ray generator worked at a power of 40 kV and 25 mA, and the patterns were collected in the angular range from 10° to 80° with 0.03° of step size. In addition, the XPS characterization of the phosphate rock samples was performed using the instrument model Thermo Scientific K-Alpha+, the monochromatic Al K*α* was the excitation source, and the energy was 1486.6 eV.

## 3. Results and Discussion

### 3.1. TPO-IR Analysis of Sulfur Species with Known Structures

**Figure 3:**
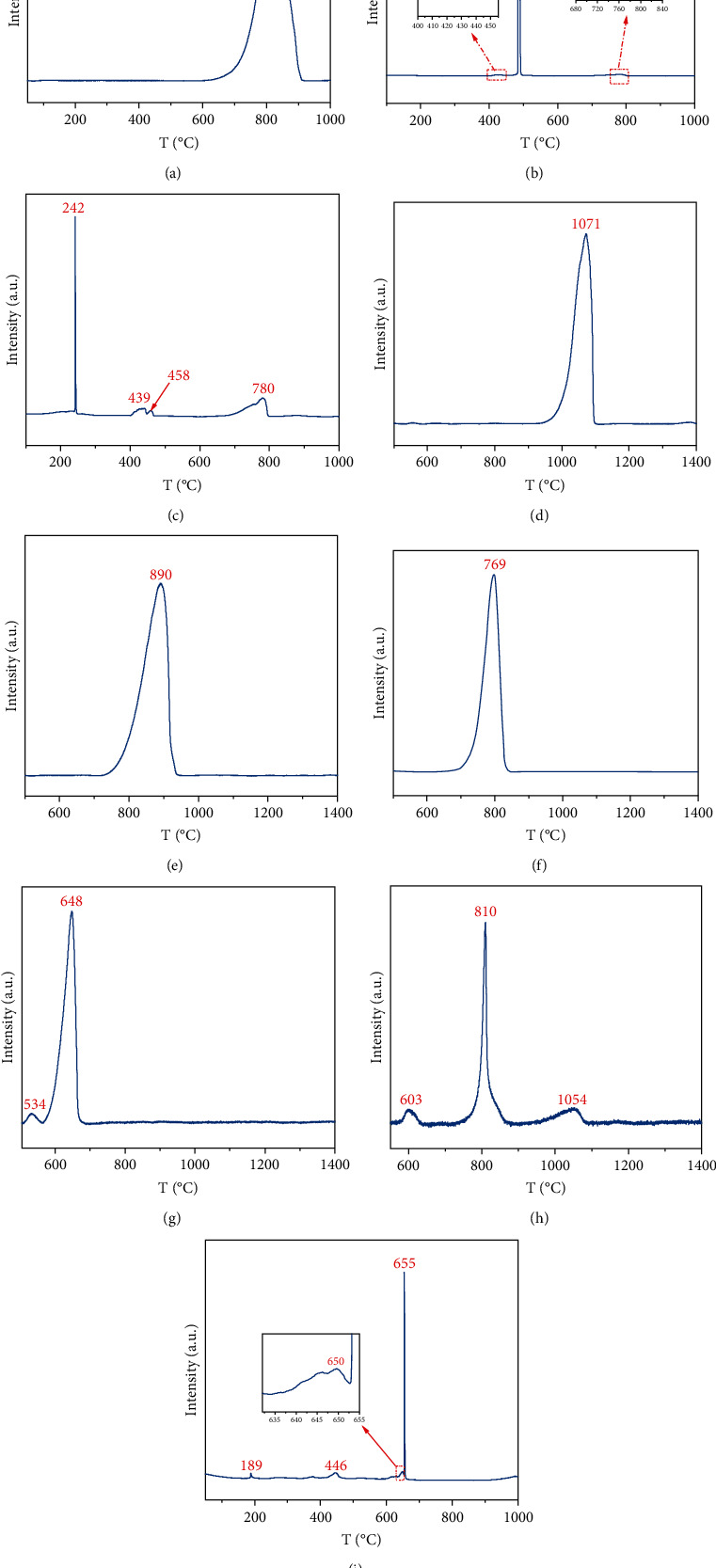
TPO-IR curve of sulfur species with known structures: (a) ZnS; (b) CoS; (c) Cu_2_S; (d) MgSO_4_·7H_2_O; (e) MnSO_4_; (f) NiSO_4_·6H_2_O; (g) FeSO_4_·7H_2_O; (h) (NH_4_)2S_2_O_8_; (i) C_18_H_29_NaO_3_S.

Figure 3 gives partial typical examples of TPO-IR curves for sulfur species with known structures. Taking ZnS as an example (Figure 3(a)), the TPO-IR curve starts to present an increasing trend at 600°C and increases abruptly after 700°C until reaching a peak at 820°C. Then, it decreases sharply and returns to the baseline position at about 900°C. This phenomenon is because of the intense SO_2_ emission peak due to the severe oxidation of ZnS at 820°C (the reaction is as shown in formula 3), so the peak temperature (*T*_max_) of 820°C is the characteristic temperature of ZnS. Similarly, the curve of MgSO_4_·7H_2_O (Figure 3(d)) clearly indicates the presence of only one peak, and the *T*_max_ of 1071°C can be identified as the characteristic temperature of MgSO_4_·7H_2_O, the relevant reaction shown in formula (4). However, typical for CoS (Figure 3(b)) and FeSO_4_·7H_2_O (Figure 3(g)), except for the two maximums intensity peaks of 486°C and 648°C, respectively, there are also several low-intensity peaks in the TPO-IR curves. It can be explained that the samples contain some impurities of other sulfur species. The corresponding chemical reaction formulas are (5) and (6), respectively. In conclusion, the *T*_max_ of maximum intensity is the characteristic temperature of sulfur reference materials.  Table 2 summarizes the *T*_max_ of the currently completed sulfur reference materials, in which T_maxi_ (*i* = 1, 2,…, 6) indicates the temperature when different characteristic peaks appear in a sample, and Arabic numerals represent the order of occurrence of characteristic peaks. *T*_max_ with “^*∗*^” indicates the characteristic temperature of the sulfur species corresponding to the chemical structure.

(3) $$\begin{array}{c} 2\text{ZnS}+3{\text{O}}_{2}⟶2\text{ZnO}+2{\text{SO}}_{2} \end{array}$$

(4) $$\begin{array}{c} 2{\text{MgSO}}_{4}\cdot 7{\text{H}}_{2}\text{O}+{\text{O}}_{2}⟶2\text{MgO}+2{\text{SO}}_{2}+7{\text{H}}_{2}\text{O} \end{array}$$

(5) $$\begin{array}{c} 2\text{CoS}+3{\text{O}}_{2}⟶2\text{CoO}+2{\text{SO}}_{2} \end{array}$$

(6) $$\begin{array}{c} 4{\text{FeSO}}_{4}\cdot 7{\text{H}}_{2}\text{O}+O_{2}⟶2{\text{Fe}}_{2}{\text{O}}_{3}+4{\text{SO}}_{2}+7{\text{H}}_{2}\text{O} \end{array}$$

### 3.2. TPO-IR Analysis of Phosphate Rock

**Figure 4:**
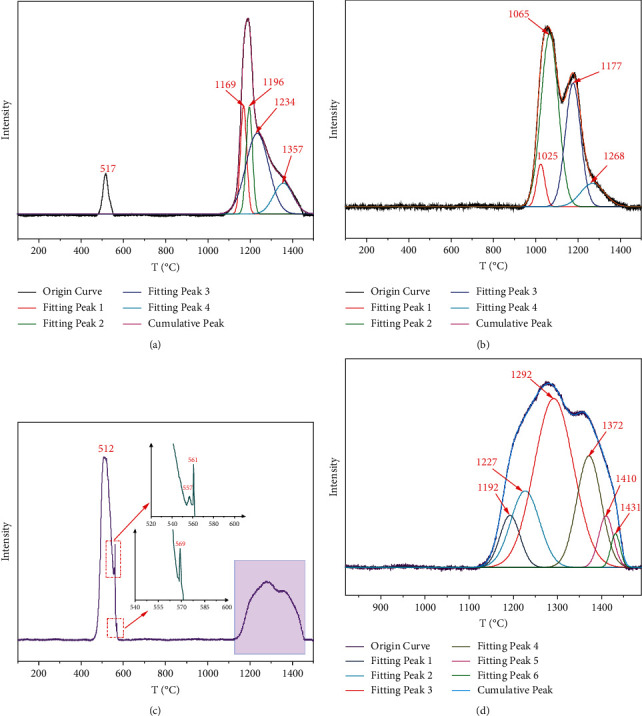
TPO-IR profiles of WAP (a), ZJP (b), and KYP (c), and locally magnified KYP (d).

Figure 4 shows the TPO-IR profiles of phosphate rock samples, and Table 3 shows the statistics of sulfur species in phosphate rock samples. Overall, the total sulfur content of KYP, WAP, and ZJP accounts for a small proportion in phosphate rocks, and the types of sulfur species are complex. As it can be seen from the curve fitting the results of KYP (Figures 4(c) and 4(d)), it has the most complex sulfur species composition with about 10 species, but the total sulfur content is only 2.14% (Table 3). Interestingly, there exists one sulfur species in the low-temperature area (<1000°C) and the mass percentage of this sulfur species is the largest compared to the others. Furthermore, as it can be seen on the enlarged curves, there are three extremely low-intensity *T*_max_ (557°C, 561°C, and 569°C) that cannot be attributed at present because the sulfur species with the known structure database is incomplete. Similar to KYP, WAP (Figure 4(a)) has a *T*_max_ of 512°C in the low-temperature area, but with much lower intensity than KYP. In the high-temperature region of more than 1000°C, WAP has about 5 sulfur species with the total sulfur content of 1.06% (Table 3). However, only 4 sulfur species with the total sulfur content of 1.18% (Table 3) in the high-temperature region (>1000°C) are observed for ZJP (Figure 4(b)). These results clearly show the similarities and differences of sulfur species in the three phosphate rock samples.

By comparing the *T*_max_ of phosphate rock samples with the sulfur reference materials, the attribution of certain sulfur species in each type of phosphate rock can be inferred. In KYP, three kinds of sulfur species can be roughly identified. Sulfur species with *T*_max_ of 512°C can be regarded as FeS (characteristic temperature is around 513°C, Table 2), and sulfur species with *T*_max_ of 1292°C can be regarded as CaSO_4_ (characteristic temperature of CaSO_4_·2H_2_O is around 1290°C, Table 2). The proportion of FeS and CaSO_4_ in the total mass is 9.22‰ and 5.62‰, respectively. The sulfur species corresponding to *T*_max_ of 1065°C and 1268°C in ZJP may be MgSO_4_ (the characteristic temperature of MgSO_4_·7H_2_O is around 1071°C, Table 2) and CaSO_4_, with contents of 6.10‰ and 1.14‰, respectively. The WAP also contains FeS and CaSO_4_, and the corresponding *T*_max_ is 517°C and 1234°C with the mass proportions of 0.64‰ and 4.82‰, respectively. In summary, it can be speculated that CaSO_4_ is distributed in all phosphate samples, KYP and WAP account for the largest proportion, while MgSO_4_ is the main sulfur species in ZJP. According to the metallogenic strata age of each phosphate rock, KYP and WAP belong to the Sinian Doushantuo formation, while ZJP belongs to the Cambrian Niutitang formation, thus, the metallogenic age and metallogenic strata can be used as strong support for the abovementioned sulfur species analysis results.

More than thirty years ago, some scholars proposed that the sulfur in the sedimentary apatite in central Guizhou came from the sulfate and bio-sulfide in seawater during the mineralization period [29,30], while the sulfur in the ocean mainly comes from volcanic hydrothermal fluids, fluid weathering, pyrite, evaporative mineral sulfur, etc. The main ways of sulfur transformation are sulfate reduction, sulfide oxidation, and sulfur disproportionation [31]. Sulfur plays an indispensable role in the process of phosphorus accumulating into an ore. The release and accumulation of phosphorus are caused by the activities of sulfate-reducing bacteria and related sulfide-oxidizing bacteria [32, 33]. Some studies have also proposed that sulfur species exist in phosphate rock in the following three forms: (1) as part of the organic matter in phosphate rocks; (2) in the form of a single mineral pyrite and sulfate in the apatite lattice; (3) in the form of SO_4_^2-^ ions [31, 34, 35]. The sulfate in KYP and WAP is dominated by CaSO_4_, while that in ZJP is dominated by MgSO_4_. But the FeS only exists in KYP and WAP. All the abovementioned phenomena can be attributed to the fact that the metallogenic periods of KYP and WAP are different from those of ZJP, and the former two are earlier. The difference in the mass proportion of FeS in KYP (9.22‰) and WAP (0.64‰) is due to the different metallogenic environments of the two phosphate ores. KYP is formed in the suboxidative zone, while WAP is formed in the oxidation zone [36, 37].

### 3.3. XRD, SEM, EDS, and XPS Analysis of Phosphate Rock

To better understand the application advantages of TPO-IR technology in the analysis of the sulfur occurrence state and genesis of phosphate rock, the phosphate rock samples were analyzed by XRD, SEM, EDS, and XPS.

**Figure 5:**
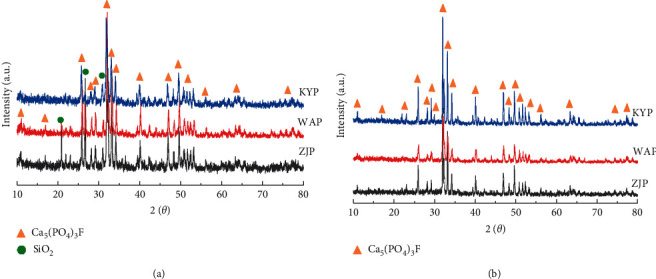
XRD patterns of phosphate rocks: (a) original sample; (b) after TPO-IR tests.

The XRD patterns of the phosphate rock samples before and after TPO-IR tests are given in Figure 5. The presence of Ca_5_(PO_4_)_3_F in all samples before and after the TPO-IR test indicates that the major components in the phosphate rocks are very stable below 1500°C, typically the characteristic diffraction angles at 25.6^o^, 31.8^o^, 32.9^o^, 34^o^, 40^o^, 46.7^o^, 49.5^o^, 53^o^, and 56^o^ were found. The characteristic diffraction peaks of silicate appear only before the TOP-IR tests, and the typical diffraction angles are distributed at 21^o^, 26.5^o^, and 30.1^o^. The intensity of the silicate peak is the most pronounced for ZJK, which suggests that ZJK has more silicate content compared to the others. The characteristic diffraction peaks of silicate disappeared after the TPO-IR tests, presumably because the silicate in the samples decomposes or transforms into an amorphous form within 1500^o^C. Unfortunately, XRD did not detect sulfur species in phosphate rocks, possibly because the content of sulfur species was below the detection limit of XRD.

**Figure 6:**
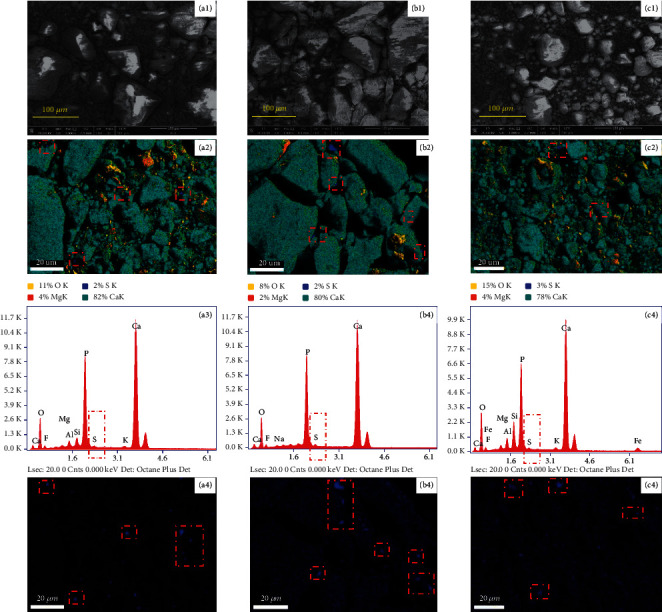
SEM and EDS images of phosphate rocks: (a) KYP series; (b) WAP series; (c) ZJP series; the number 1 represents SEM images, while 2, 3, and 4 represent EDS results.

Figure 6 shows the SEM and EDS images of the phosphate rock samples. The series a, *b*, and *c* represent the images of KYP, WAP, and ZJP, respectively. From Figure 6(a1, b1, and c1), it can be seen that the particle sizes of the selected samples are basically the same within a range of 5–120 *μ*m. It can be seen from Figure 6(a2, b2, and c2) and Figure 6(a4, b4, and c4) that the sulfur content in the phosphate rock is very small and has the characteristics of local enrichment (blue pixels represent elemental sulfur in phosphate rock, marked with red rectangles).

Combined with the element intensities in Figure 6(a3, b3, and c3), Ca, P, O, Si, and F account for the largest proportion in the phosphate rock samples, while S only accounts for a small proportion (marked with a red rectangle). Therefore, the content and distribution of sulfur in phosphate rock samples can be determined by SEM and EDS characterization methods, but the specific sulfur species cannot be determined. Moreover, the proportion of sulfur is about 2% for KYP and WAP, and 3% for ZJP, while the test results of TPO-IR show that sulfur in KYP, WAP, and ZJP accounted for 2.14%, 1.06%, and 1.18%, respectively. In general, the difference between TPO-IR data and EDS results can be explained by the fact that EDS is a semiquantitative analysis method for element analysis in the microregion, while the quantitative analysis of TPO-IR is accurate and belongs to macro analysis. Therefore, the analysis results of TPO-IR are more reliable in quantitative analysis of sulfur species.

**Figure 7:**
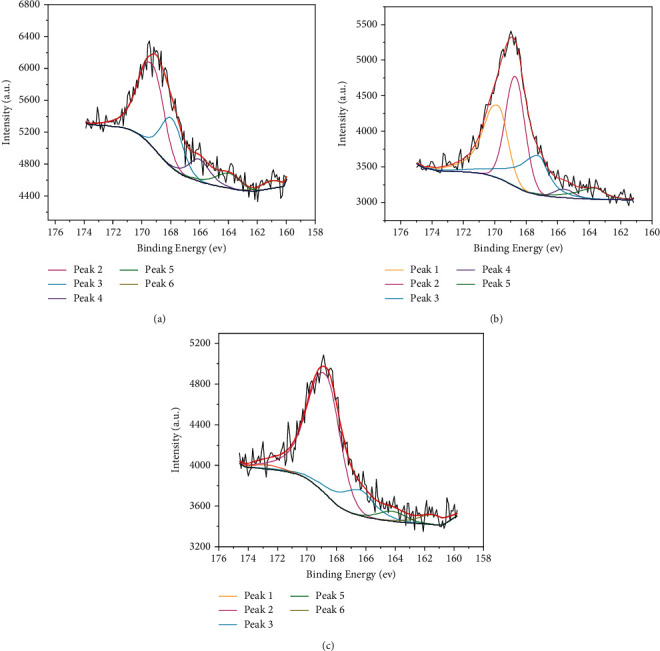
The XPS S 2p fitting curves: (a) ZJP, (b)WAP, and (c) KYP.

XPS can analyze the surface elements (except H and He) and their existence modes on the sample according to the difference of the electron binding energy and the valence state of different elements in the photoelectron, and then infers the composition and chemical structure of the sample. The Thermo Fisher Avantage special software was used to perform peak fitting on the obtained S 2p XPS spectrum of phosphate rocks, and the fitting parameters were set as Shirley Background and 0% Lorentzian-Gaussian, as shown in Figure 7. It is found that the existing forms of sulfur in KYP, WAP, and ZJP can be basically divided into six types. The results are summarized in Table 4. The peaks 2 and 3 near 168.9 and 167.4 eV are caused by sulfate and sulfite, respectively. It can be seen that the sulfate and sulfite appear in all phosphate rock samples and account for a large proportion, the relative contents of sulfate in the three phosphate rocks are 70.92 mol.% of KYP, 35.26 mol.% of WAP, and 51.24 mol.% of ZJP, respectively. It can be inferred that the sulfur on the surface of phosphate rocks is mainly composed of sulfate and sulfite, especially the sulfate which accounts for the largest proportion. The binding energy positions of peak 1 (172.3 eV), peak 4 (166.4 eV), peak 5 (164.3 eV), and peak 6 (161.5 eV) can be classified as pyrite, sulfoxide, thiophene, and sulfide, respectively. According to XPS results, the sulfur species in the phosphate rocks exist in the form of sulfate, sulfite, sulfide, and organic sulfur. Although XPS only gets the sulfur species information on the sample surface, while TPO-IR obtains the sulfur species information of the sample bulk phase, the results are roughly the same. Both XPS and TPO-IR indicate that the sulfur species in the phosphate rocks were mainly sulfates. In fact, similar to EDS, XPS is also semiquantitative, but TPO-IR is quantitative, and the detection results of TPO-IR are more accurate.

## 4. Conclusion

By improving TPO-IR technology and deeply mining data, more accurate detection results of sulfur species were obtained than previous works. TPO-IR results show that the total sulfur contents of the three phosphate rocks were 2.14% for KYP, 1.18% for ZJP, and 1.06% for WAP, respectively. And KYP and WAP are mainly CaSO_4_, with the content of 5.62‰ and 4.82‰, respectively, while ZJP is mainly MgSO_4_ with the content of 6.1‰. In addition, both KYP and WAP contain FeS with a content of 9.22‰ and 0.64‰, respectively, but not in ZJP. It is related to the metallogenic age, which reflects that the oxidation strength of the ZJP metallogenic environment is higher than that of KYP and WAP. Compared with semiquantitative EDS and XPS, it is found that TPO-IR technology only needs to establish a standard database of characteristic temperature of sulfur species, so that sulfur species in phosphate rocks can be easily characterized and quantified. Since the TPO-IR technology can qualitatively and quantitatively detect sulfur species in minerals, it can facilitate the development of environmental monitoring, mineral exploration, mining, beneficiation, and sulfur-related chemical industries. Furthermore, this study also provides a theoretical basis and experimental support for the application of TPO-IR technology for the detection of sulfur in other solid minerals.

## Figures and Tables

**Table 1:** Physical properties of sulfur species with known structures used in this work.

Number	Samples	Properties		
		Molecular weight	Melting point (°C)	Purity (%)
1	Cu_2_S	159.16	1100–1130	99.000
2	MnS	87.00	1610	98.000
3	FeS	87.91	1195	99.000
4	WS_2_	247.97	1489	99.900
5	CoS	97.00	1116	99.500
6	ZnS	97.46	1700	99.999
7	Ag_2_S	247.80	825	99.995
8	CrK(SO_4_)·12H_2_O	403.00	89	—
9	NiSO_4_·6H_2_O	262.96	31.5	—
10	(NH_4_)_2_S_2_O_8_	228.20	120	≥98
11	Na_2_SO_3_	126.04	150	≥97
12	H_2_N·NH_2_·H_2_SO_4_	130.12	254	≥99
13	(NH_4_)_2_SO_4_	132.14	235–280	≥99
14	MnSO_4_	169.02	700	99.000
15	MgSO_4_·7H_2_O	246.47	1124	99.000
16	FeSO_4_·7H_2_O	278.02	64	99–101
17	CaSO_4_·2H_2_O	172.17	1450	≥99
18	C_18_H_29_NaO_3_S	348.48	>300	—

**Table 2:** *T*_max_ of the currently completed sulfur reference materials.

Sample	Formula	Oxidation temperature of sulfur species					
		*T*_max1_/°C	*T*_max2_/°C	*T*_max3_/°C	*T*_max4_/°C	*T*_max5_/°C	*T*_max6_/°C
Sulfide	Ag_2_S	554^*∗*^	634	652	678	918	941
	MnS	265	310	335	400	570	868^*∗*^
	FeS	230	513^*∗*^	655	933	961	—
	Cu_2_S	242^*∗*^	439	458	780	—	—
	WS_2_	370	414	438	496^*∗*^	—	—
	CoS	423	486^*∗*^	783	—	—	—
	ZnS	820^*∗*^	—	—	—	—	—

Sulfate	CaSO_4_·2H_2_O	1290^*∗*^	1315	1322	1333	1349	1352
	H_2_NNH_2_H_2_SO_4_	265	281	282	283^*∗*^	398	—
	CrK(SO_4_)·12H_2_O	746^*∗*^	986	1048	—	—	—
	Na_2_SO_3_	1094^*∗*^	1197	1300	—	—	—
	(NH_4_)_2_S_2_O_8_	603	810^*∗*^	1054	—	—	—
	FeSO_4_·7H_2_O	534	648^*∗*^	—	—	—	—
	MgSO_4_·7H_2_O	1071^*∗*^	—	—	–	—	—
	MnSO_4_	890^*∗*^	—	—	—	—	—
	(NH_4_)_2_SO_4_	350^*∗*^	—	—	—	—	—
	NiSO_4_·6H_2_O	796^*∗*^	—	—	—	—	—

Organic sulfur	C_18_H_29_NaO_3_S	189	446	655^*∗*^	—	—	—

The symbol ‘—' indicates no peaks appear at this temperature.

**Table 3:** Statistics of sulfur species of phosphate rock samples.

Sample	Statistics of sulfur species					
	*T*_max_/^o^C	*M*_s_/(^*∗*^10^−3^ g)	*M*_1_/(^*∗*^10^−3^ g)	S%	*M*_i_/(^*∗*^10^−3^ g)	S‰
KYP	512	412.5	8.838	2.14	3.8025	9.22
	557				0.0957	0.23
	561				0.1476	0.36
	569				0.0077	0.02
	1192				0.3652	0.89
	1227				0.7709	1.87
	1292				2.3184	5.62
	1372				0.9552	2.32
	1410				0.265	0.64
	1431				0.1098	0.27

ZJP	1025	420.79	4.9811	1.18	0.3044	0.72
	1065				2.5647	6.10
	1177				1.6309	3.88
	1268				0.4812	1.14

WAP	517	504.37	5.3244	1.06	0.3239	0.64
	1169				0.8924	1.77
	1196				0.8832	1.75
	1234				2.4331	4.82
	1357				0.7917	1.57

**Table 4:** The XPS S 2p analysis of phosphate rocks.

Peak	Assignment	Position/eV	F_whm_/eV	Content (mol.%)		
				KYP	WAP	ZJP
1	Pyrite	172.3	1.9	1.94	32.43	0
2	Sulfate	168.9	1.9	70.92	35.26	51.24
3	Sulfite	167.4	1.9	17.18	23.33	24.38
4	Sulfoxide	166.4	1.9	0	2.73	11.47
5	Thiophene	164.3	1.9	4.81	6.25	9.09
6	Sulfide	161.5	1.9	5.15	0	3.82

## Data Availability

The data that support the findings of this study are available from the corresponding author upon request.

## References

[1] Prasad R., Shivay Y. S. (2018). Sulphur in soil, plant and human nutrition. *Proceedings of the National Academy of Sciences, India - Section B: Biological Sciences*.

[2] Labidi J., Shahar A., Le Losq C., Hillgren V., Mysen B., Farquhar J. (2016). Experimentally determined sulfur isotope fractionation between metal and silicate and implications for planetary differentiation. *Geochimica et Cosmochimica Acta*.

[3] Farquhar J., Wing B. A. (2003). Multiple sulfur isotopes and the evolution of the atmosphere. *Earth and Planetary Science Letters*.

[4] Mcguire M. M., Jallad K. N., Ben-Amotz D., Hamers R. J. (2001). Chemical mapping of elemental sulfur on pyrite and arsenopyrite surfaces using near-infrared Raman imaging microscopy. *Applied Surface Science*.

[5] Hofmann A., Bekker A., Rouxel O., Rumble D., Master S. (2009). Multiple sulphur and iron isotope composition of detrital pyrite in Archaean sedimentary rocks: a new tool for provenance analysis. *Earth and Planetary Science Letters*.

[6] LaFlamme C., Sugiono D., Thébaud N. (2018). Multiple sulfur isotopes monitor fluid evolution of an Archean orogenic gold deposit. *Geochimica et Cosmochimica Acta*.

[7] Gu Y., Yperman J., Vandewijngaarden J., Reggers G., Carleer R. (2017). Organic and inorganic sulphur compounds releases from high-pyrite coal pyrolysis in H2, N2 and CO2: test case Chinese LZ coal. *Fuel*.

[8] Liu G., Peng Z., Yang P., Wang G. (2001). Sulfur in coal and its environmental impact from yanzhou mining district, China. *Chinese Journal of Geochemistry*.

[9] Chou C. L. (2012). Sulfur in coals: a review of geochemistry and origins. *International Journal of Coal Geology*.

[10] Edwards K. J., Bond P. L., Banfield J. F. (2000). Characteristics of attachment and growth of Thiobacillus caldus on sulphide minerals: a chemotactic response to sulphur minerals?. *Environmental Microbiology*.

[11] Baioumy H. (2011). Rare earth elements and sulfur and strontium isotopes of upper Cretaceous phosphorites in Egypt. *Cretaceous Research*.

[12] Berndmeyer C., Birgel D., Brunner B. (2012). The influence of bacterial activity on phosphorite formation in the Miocene Monterey Formation, California. *Palaeogeography, Palaeoclimatology, Palaeoecology*.

[13] He G., Zhou Y. (2015). Geology of phosphate rock in China: distribution, rock type and metallogenic perspective. *Global Environmental Research*.

[14] Zhang W., Ma W., Zhang F. S., Ma J. (2005). Comparative analysis of the superiority of China’s phosphate rock and development strategies with that of the United States and Morocco. *Journal of Natural Resources*.

[15] Pérez-Cabero M., Rodríguez-Ramos I., Guerrero-Ruz A. (2003). Characterization of carbon nanotubes and carbon nanofibers prepared by catalytic decomposition of acetylene in a fluidized bed reactor. *Journal of Catalysis*.

[16] Gonzalez D., Altin O., Eser S., Garcia A. B. (2007). Temperature-programmed oxidation studies of carbon materials prepared from anthracites by high temperature treatment. *Materials Chemistry and Physics*.

[17] Aso H., Matsuoka K., Sharma A., Tomita A. (2004). Evaluation of size of graphene sheet in anthracite by a temperature-programmed oxidation method. *Energy & Fuels*.

[18] Eser S., Venkataraman R., Altin O. (2006). Deposition of Carbonaceous solids on different substrates from thermal stressing of JP-8 and jet a fuels. *Industrial & Engineering Chemistry Research*.

[19] Rey A., Zazo J. A., Casas J. A., Bahamonde A., Rodriguez J. (2011). Influence of the structural and surface characteristics of activated carbon on the catalytic decomposition of hydrogen peroxide. *Applied Catalysis A: General*.

[20] Xie J., Sharma P. K., Varadan V. V., Varadan V., Pradhan B. K., Eser S. (2002). Thermal, Raman and surface area studies of microcoiled carbon fiber synthesized by CVD microwave system. *Materials Chemistry and Physics*.

[21] Steger H. F. (1976). Determination of the elemental sulphur content of minerals and ores. *Talanta*.

[22] Wilkin R. T., Bischoff K. J. (2006). Coulometric determination of total sulfur and reduced inorganic sulfur fractions in environmental samples. *Talanta*.

[23] Jin B. H. (2002). Petrographic analysis and classical method. *Rock and Mineral Analysis*.

[24] Kelemen S. R., George G. N., Gorbaty M. L. (1990). Direct determination and quantification of sulphur forms in heavy petroleum and coals: 1. The X-ray photoelectron spectroscopy (XPS) approach. *Fuel*.

[25] Fabbi B. P., Moore W. J. (1970). Rapid X-ray fluorescence determination of sulfur in mineralized rocks from the bingham mining district, Utah. *Applied Spectroscopy*.

[26] Bolin T. B., Birdwell J. E., Lewan M. D. (2016). Sulfur species in source rock bitumen before and after hydrous pyrolysis determined by X-ray absorption near-edge structure. *Energy & Fuels*.

[27] Tang S., Yang S., Tan S., Guo J., Tang A. (2020). Fast determination of the sulfur species in solid phase systems by a tpo-ir method. *Quím Nova*.

[28] Zhang Y. G. (2019). Three stages dynamic mineralization model of phosphorus-rich ore: the mechanism of high-grade phosphate ore formation in Kaiyang style, Qianzhong. *Journal of Palaeogeography*.

[29] Chu X., Feng L., Chen Q. (1995). Sulfur isotope and its significance of the late sinian phosphates in Kaiyang district, Guizhou province. *Science Bulletin*.

[30] Chen Q., Feng L. (1996). Sulphur and carbon isotopes of sedimentary apatite, Guizhou province and their geological significance. *Acta Petrologica Sinica*.

[31] Wu N., Farquhar J., Strauss H. (2014). *δ*34S and Δ33S records of Paleozoic seawater sulfate based on the analysis of carbonate associated sulfate. *Earth and Planetary Science Letters*.

[32] Arning E. T., Birgel D., Brunner B., Peckmann J. (2009). Bacterial formation of phosphatic laminites off Peru. *Geobiology*.

[33] Wang J., Shen S., Kang J., Li H., Guo Z. (2010). Effect of ore solid concentration on the bioleaching of phosphorus from high-phosphorus iron ores using indigenous sulfur-oxidizing bacteria from municipal wastewater. *Process Biochemistry*.

[34] Benmore R. A., Coleman M. L., Mcarthur J. M. (1983). Origin of sedimentary francolite from its sulphur and carbon isotope composition. *Nature*.

[35] Nathan Y., Nielsen H. (1980). *Sulfur isotopes in phosphorites*.

[36] Yang H., Xiao J., Xia Y. (2019). Origin of the ediacaran weng’an and Kaiyang phosphorite deposits in the nanhua basin, SW China. *Journal of Asian Earth Sciences*.

[37] Ray Liu Z. R., Zhou M. F. (2017). Meishucun phosphorite succession (SW China) records redox changes of the early Cambrian ocean. *The Geological Society of America Bulletin*.

